# Probing the ArcA regulon under aerobic/ROS conditions in *Salmonella enterica* serovar Typhimurium

**DOI:** 10.1186/1471-2164-14-626

**Published:** 2013-09-17

**Authors:** Eduardo H Morales, Bernardo Collao, Prerak T Desai, Iván L Calderón, Fernando Gil, Roberto Luraschi, Steffen Porwollik, Michael McClelland, Claudia P Saavedra

**Affiliations:** 1 Laboratorio de Microbiología Molecular, Facultad Ciencias Biológicas, Universidad Andres Bello, Santiago, Chile; 2 Department of Microbiology and Molecular Genetics, B240 Medical Sciences Building, University of California, Irvine, CA 92697, USA; 3 Present address: Great Lakes Bioenergy Research Center, University of Wisconsin-Madison, Madison, Wisconsin, USA; 4 Present address: Department of Biomolecular Chemistry, University of Wisconsin-Madison, Madison, Wisconsin, USA

**Keywords:** ArcAB two-component system, Oxidative stress, Hydrogen peroxide resistance

## Abstract

**Background:**

Hydrogen peroxide (H_2_O_2_) is a reactive oxygen species (ROS), which is part of the oxidative burst encountered upon internalization of *Salmonella enterica* serovar Typhimurium (*S*. Typhimurium) by phagocytic cells. It has previously been established that, the ArcAB two-component system plays a critical role in ROS resistance, but the genes regulated by the system remained undetermined to date. We therefore investigated the ArcA regulon in aerobically growing *S*. Typhimurium before and after exposure to H_2_O_2_ by querying gene expression and other physiological changes in wild type and Δ*arcA* strains.

**Results:**

In the Δ*arcA* strain, expression of 292 genes showed direct or indirect regulation by ArcA in response to H_2_O_2_, of which 141were also regulated in aerobiosis, but in the opposite direction. Gene set enrichment analysis (GSEA) of the expression data from WT and Δ*arcA* strains, revealed that, in response to H_2_O_2_ challenge in aerobically grown cells, ArcA down regulated multiple PEP-PTS and ABC transporters, while up regulating genes involved in glutathione and glycerolipid metabolism and nucleotide transport. Further biochemical analysis guided by GSEA results showed that deletion of *arcA* during aerobic growth lead to increased reactive oxygen species (ROS) production which was concomitant with an increased NADH/NAD^+^ ratio. In absence of ArcA under aerobic conditions, H_2_O_2_ exposure resulted in lower levels of glutathione reductase activity, leading to a decreased GSH (reduced glutathione)/GSSG (oxidized glutathione) ratio.

**Conclusion:**

The ArcA regulon was defined in 2 conditions, aerobic growth and the combination of peroxide treatment and aerobic growth in *S*. Typhimurium. ArcA coordinates a response that involves multiple aspects of the carbon flux through central metabolism, which ultimately modulates the reducing potential of the cell.

## Background

*Salmonella enterica* serovar Typhimurium (*S.* Typhimurium) is a Gram-negative, facultative anaerobe and intracellular bacterium that causes gastroenteritis, bacteremia and enteric fever in the murine model [1]. During its infective cycle, *S*. Typhimurium is internalized by phagocytes where it is exposed to a series of antimicrobial compounds including reactive oxygen species (ROS) which trigger the production of superoxide (O_2_^-^) by phagocytic NADPH oxidase. O_2_^-^ is unstable with a half life in the order of milliseconds, and under acidic conditions, as those found within the *Salmonella* containing vacuole (SCV), two molecules of O_2_^-^ react to generate H_2_O_2_[2]. Additionally, the *S*. Typhimurium genome codes for both cytoplasmic (*sodA* and *sodB*) and periplasmic (*sodCI* and *sodCII*) superoxide dismutases, that catalyze the generation of H_2_O_2_ and molecular oxygen from O_2_^-^[3-5].

The response of the bacterium to H_2_O_2_ has been mostly related to the transcription factor OxyR [6], however, several studies in *Escherichia coli* (*E. coli*), *S*. Typhimurium, *S*. Enteritidis and *Haemophilus influenzae* indicate that the response regulator ArcA is required for H_2_O_2_ resistance [7-10]. Furthermore, in *E. coli* H_2_O_2_ resistance depends on the cognate sensor ArcB [7]. The ArcAB two-component system is composed of the response regulator ArcA and the hybrid sensor kinase ArcB [11,12]. ArcAB responds to shifts in oxygen concentration [13,14], however, the signal that activates the system remains elusive since some studies show no correlation between ArcAB activity and the ubiquinone pool [13], while others indicate that the system’s activity depends on the ubiquinone and/or menaquinone pools [15-17]. Upon reduction of the redox-active cysteine residues between two monomers, ArcB undergoes autophosphorylation in an ATP-dependent intramolecular reaction at a conserved histidine residue located at position 292 [18,19]. The signal is transferred to residues D576 and H717 of ArcB and finally to residue D54 of ArcA [12,19,20]. Phosphorylated ArcA (ArcA-P) forms a tretamer of dimers in a 1:1 ratio of ArcA and ArcA-P, which binds to promoter regions, thereby regulating gene expression [21,22].

Several studies in *E. coli*, *S*. Typhimurium, *H. influenzae* and *Shewanella oneidensis* have used global gene expression profiling to determine the ArcA regulon under anaerobic conditions, showing that the effect of ArcA is pleiotropic and coordinates a response that includes changes in cellular metabolism, motility and chromosomal replication, among others [8,10,23-26]. In *S*. Typhimurium 14028s grown under anaerobic conditions, ArcA regulates either directly or indirectly more than 392 genes. Additionally, an *arcA* mutant has a longer doubling time than the wild type strain, lacks flagella, is non-motile and remains fully virulent [8].

In contrast to the vast amount of information about the role of ArcA in anaerobiosis, little is known about the genes or biochemical processes that ArcA regulates in response to H_2_O_2_. Previous studies have mainly shown that *arcA* or *arcB* mutant strains are more sensitive to the toxic compound [8-10]. One study conducted in *E. coli* used a proteomic approach and determined that ArcA regulates the expression of *fliC*, *oppA* and *gltI* in response to H_2_O_2_[7], while in *S*. Typhimurium ArcA negatively regulates *ompD* and *ompW*[27,28]. To gain further insights into the role of ArcA in ROS resistance, we compared transcriptional changes in *S.* Typhimurium 14028s wild-type and Δ*arcA* strains with and without peroxide exposure under aerobic conditions. As expected, the Δ*arcA* mutation affected multiple pathways confirming that ArcA has a pleiotropic effect and plays a role as a global regulator. Interestingly, the genes regulated by ArcA in response to H_2_O_2_ differ from those regulated under anaerobic conditions [8]. A Gene Set Enrichment Analyses using the KEGG database predicted that 10 pathways were up-regulated and 2 down-regulated by ArcA in response to H_2_O_2_ treatment in aerobiosis. Finally, biochemical analyses showed that under aerobic conditions ArcA modulates the redox potential of the cell by regulating the levels of NADH and of intracellular ROS. After H_2_O_2_ exposure under aerobic conditions, ArcA was found to regulate turnover of reduced glutathione (GSH).

## Methods

### Bacterial strains and growth conditions

Pre-cultures of strains 14028s wild type and Δ*arcA* were streaked from cryo-vials stored at −80°C onto LB agar plates and allowed to grow at 37°C for 12 h. One colony was picked and grown in a 250 ml Erlenmeyer flask containing 25 ml of LB broth for 16 h at 37°C on a rotary shaker at 200 rpm. Exactly 500 μl of the cultures were then transferred into 500 ml Erlenmeyer flasks containing 50 ml of LB broth and grown in a temperature controlled rotary shaker at 200 rpm (LSI-3016R, Labtech Shaking Incubator, Indonesia). Optical density (OD_600_) was measured until reaching the desired OD_600_ for treatment with H_2_O_2_ (~ 0.4, corresponding to an incubation time of about 2.5 h). These conditions closely resemble those used in a previous study with *E. coli,* where 50 ml of culture grown at 200 rpm in a shaking incubator at 37°C to an OD_546_ of ~ 0.4 exhibited a pO_2_ of ≥ 90% [29]. Solid media contained agar (20 g l^-1^), and plates were incubated at 37°C. When necessary, growth media was supplemented with the appropriate antibiotics.

### Microarray analysis

Overnight cultures of strains 14028s and Δ*arcA* were diluted (1:100) and cells were grown to OD_600_ ~ 0.4 as described. At this point, H_2_O_2_ (1 mM) was added and cells were grown for 20 min. Control cells received no treatment. Experiments were performed in triplicate on different days. After exposure to the toxic compound, 5 ml of ice cold 5% (v/v) phenol pH 4.3 / 95% (v/v) ethanol was added to 25 ml of culture and left on ice for 20 min. Subsequently, 8 ml of this solution were centrifuged for 10 min at 8000 rpm, the supernatant was removed and the bacterial pellet was resuspended with 200 μl of 10 mM Tris–HCl (pH 8.0) that included 4 μl of lysozyme (50 mg/ml). The reaction was incubated for 10 min at 37°C, and total RNA was extracted using the High Pure RNA Isolation kit (Roche) following the manufacturer’s instructions. RNA was eluted in 105 μl of water and treated with DNaseI (Roche) at 37°C for 30 min. Total RNA was recovered using the Qiagen RNeasy kit (Qiagen), following the manufacturer’s instructions. RNA was eluted in 80 μl and subjected to a second round of DNaseI treatment (Ambion Turbo DNA-free kit) at 37°C for 30 min, purified, recovered using the Qiagen RNeasy kit (Qiagen) following the manufacturer’s instructions and eluted in 55 μl of water.

Exactly 20 μg of total RNA were used for labeling with Cy3 or Cy5. Briefly, the RNA volume was adjusted to 30 μl, 2 μl of random hexamers N_6_ (Sigma, 2 μg/μl) were added and the mixture was incubated for 10 min at 70°C. Subsequently, cDNA was generated using Superscript II (Invitrogen) following the manufacturer’s instructions. Final nucleotide concentrations of the reaction were 0.5 mM dATP, dTTP, dGTP and 0.2 mM dCTP. After addition of the master mix, 4 μl of 5 mM dye labeled dCTP (Cy3 or Cy5) were added to the reaction and the mixture was incubated at 42°C for 60 min. After this time, 2 μl of Superscript II were added and the reaction was incubated at 42°C for an additional 60 min. The reaction was stopped by adding 3 μl of 1 M NaOH and incubating at 70°C for 10 min. The pH was neutralized by adding 3 μl of 1 M HCl. The labeled cDNA was purified using the Qiagen PCR purification kit following the manufacturer’s instructions. The purified labeled cDNA (4 μg) was hybridized to a ~ 387.000 50-mer NimbleGen microarray (Roche NimbleGen), tiling the *S*. Typhimurium 14028s genome at overlapping intervals of about 12 bases on both strands, as previously described [30].

### Data acquisition and analysis

Arrays were scanned using a GenePix 4000B laser scanner (Molecular Devices, Sunnyvale, California) at 5 μm resolution. Signal intensities were quantified using NimbleScan software v2.4 (Roche NimbleGen). Intensity values were background subtracted, normalized within (median) and between (quantile) the arrays using WebarrayDB [31], and converted to log_2_ values. For each array, the background was calculated as follows: log_2_ median intensity value for negative control probes + (3 * log_2_ intensity value standard deviation negative control probes). Negative control probes correspond to the probes located on the non-coding strand of each gene in the array. Genes with intensity values over the background were included in the analysis. After array data acquisition and normalization, two-way ANOVA was performed using MeV TM4 software [32], to determine uncorrected p-values. For the analysis, two categories were considered (genotype and treatment), each with two sub-categories. False Discovery Rate (FDR) adjusted q values were calculated using QVALUE in Bioconductor [33]. Genes with a q value ≤ 0.05 for interaction and a ratio of ≥ 2 between the fold change of strains 14028s wild type and Δ*arcA* ((wild type treated/wild type control)/(Δ*arcA* treated/ Δ*arcA* control)) were considered to be differentially regulated in response to H_2_O_2_. Genes with a q-value of ≤ 0.05 for genotype and a fold change of ≥ 2 between strains Δ*arcA* and wild type (Δ*arcA* control/wild type control) without treatment were considered to be differentially regulated in aerobiosis. The microarray data has been deposited in GEO (http://www.ncbi.nlm.nih.gov/geo/) and is accessible via GEO Accession Number GSE34134.

Prediction of metabolic pathways altered in the different strains by treatment with H_2_O_2_ or due to the mutation of *arcA* was performed using the software Gene Set Enrichment Analysis (GSEA) [34], with the KEGG database for *S*. Typhimurium LT2 as a reference. Briefly, GSEA is a computational method that determines whether an a priori defined set of genes shows statistically significant, concordant differences between two biological states [34]. To determine the pathways regulated by ArcA in response to H_2_O_2_, the log_2_ values of all replicas were averaged and treated as follows: (log_2_ 14028s wild type H_2_O_2_ aerobic - log_2_ 14028s wild type aerobic) - (log_2_ Δ*arcA* H_2_O_2_ aerobic - log_2_ Δ*arcA* aerobic). Positive Normalized Enrichment Score (NES) values represent pathways up-regulated by ArcA, while negative NES values represent pathways negatively regulated upon H_2_O_2_ treatment under aerobic conditions. To determine the pathways regulated under aerobic conditions, the log_2_ values of all replicas of untreated cells were averaged and treated as follows: (log_2_ Δ*arcA* aerobic - log_2_ 14028s wild type aerobic). Pathways with an FDR of ≤ 0.25 as determined by GSEA were considered to present significant changes.

### Real time quantitative RT-PCR

qRT-PCR was performed using the primers listed in Additional file 1: Table S1 as previously described [28], with a minor modification of the PCR program. Briefly, relative quantification was performed using Brilliant II SYBR Green QPCR Master Reagent Kit and the M×3000P detection system (Stratagene). 16S rRNA was used for normalization. The reaction mixture was carried out in a final volume of 20 μl containing 1 μl of diluted cDNA (1:1000), 0.24 μl of each primer (120 nM), 10 μl of 2 × Master Mix, 0.14 μl of diluted ROX (1:200) and 8.38 μl of H_2_O. The reaction was performed under the following conditions: 10 min at 95°C followed by 40 cycles of 30 s at 95°C, 30 s at 58°C and 30 s at 72°C. Finally, a melting cycle from 65°C to 95°C was performed to check for amplification specificity. Amplification efficiency was calculated from a standard curve constructed by amplifying serial dilutions of RT-PCR products for each gene. These values were used to obtain the fold-change in expression for the gene of interest normalized with 16S levels according to Pfaffl [35].

### Promoter analysis

A positional weight matrix was generated using the ArcA-binding sites predicted in *E. coli* for which footprinting experiments are available reviewed in [36]. Additionally, the binding sites predicted for members of the ArcA regulon in *S*. Typhimurium 14028s in anaerobiosis were also included [8], as was that of the *ompW* promoter region, which was shown to be functional [28]. The upstream sequences of the genes regulated by ArcA in response to aerobiosis or H_2_O_2_ exposure under aerobic conditions (Additional file 2: Table S2) were retrieved (positions −400 to −1 with respect to the translation start site) from the sequenced and annotated genome of *S*. Typhimurium 14028s [37]. Promoter regions with less than 20 nt between the translation start site of the ORF under analysis and the end or start of the upstream ORF were not included in the analysis. Binding sites at the promoter regions of genes regulated by ArcA in response to aerobiosis or H_2_O_2_ exposure under aerobic conditions (Additional file 2: Table S2) were predicted using the Matrix-scan software [38] available at http://rsat.ulb.ac.be/. The parameters used for the analysis were those given by default by the software. Binding sites with a p-value of ≤ 0.0001 were considered significant and reported as predicted ArcA binding sites.

### Biochemical determinations

Overnight cultures of strains 14028s wild type and Δ*arcA* were diluted (1:100) and cells were grown to OD_600_ ~ 0.4. At this point, H_2_O_2_ (1.0 mM) was added and cells were grown for 20 min. Control cells received no treatment. Experiments were performed in triplicate on different days. After treatment, 6 ml of cultures were withdrawn for each analysis and used for measurement of NADH, glutathione (GSH) and glutathione reductase (GR) activity. NADH was measured using commercially available kits by Abcam. The ratio between reduced glutathione and oxidized glutathione (GSH/GSSG) and GR activity were measured using commercially available kits by Cayman Chemicals. In all cases, measurements were performed following the instructions provided by the manufacturers without modifications.

Measurement of intracellular ROS was performed using the oxidant-sensitive probe H_2_DCFDA, as previously described, with minor modifications [39]. Briefly, aerobically grown cells in LB at OD_600_ ~ 0.4 were incubated with 10 μM H_2_DCFDA. At 10 min intervals aliquots were taken, washed with 10 mM potassium phosphate buffer, pH 7.0, resuspended in the same buffer, and disrupted by sonication. Cell extracts (100 μl) were mixed with 1 ml phosphate buffer and fluorescence was measured using a TECAN Infinite 200 PRO Nanoquant microplate reader (excitation, 480 nm; emission, 520 nm). Emission values were normalized based on the bacterial concentration as determined by the Optical Density (OD) of the culture at 600 nm.

## Results

To analyze the role of ArcA in the transcriptional response to aerobiosis and H_2_O_2_, the ArcA regulon of *S*. Typhimurium was determined by microarray analysis. Expression profiles were measured from three independent samples of aerobically grown wild-type (14028s) and Δ*arcA* strains with or without 1 mM H_2_O_2_. After normalization, 3949 genes showed intensity values over the background in at least one array and were included in the analysis. The results were validated by randomly selecting eight genes and measuring the transcript levels by qRT-PCR (Additional file 1: Table S1). A statistically significant correlation was observed between microarray and qRT-PCR data (r^2^ = 0.7, p-value ≤ 0.0001), despite quantitative differences in the level of change, suggesting that the results obtained by microarray analysis reflect the actual changes in gene expression.

In the wild type strain, expression of 381 and 667 genes was up- or downregulated, respectively, in response to H_2_O_2_ under aerobic conditions (fold change ≥ 2, FDR q-value treatment ≤ 0.05, Additional file 2: Table S2). Several genes known to be upregulated by H_2_O_2_ and required for its resistance were among the upregulated genes in the wild type and Δ*arcA* strains, including *katE*, *katG* and *mntH*[40]. A previous study investigated the effect of H_2_O_2_ on gene expression in a different *S.* Typhimurium strain, 4/74 [41]. That study found 309 genes to be upregulated after H_2_O_2_ exposure, and 428 genes to be downregulated. The concurrence with our results in strain 14028s is about 30%: 119/381 upregulated 14028s genes and 191/667 downregulated 14028s genes had been found to be similarly regulated in strain 4/74. The observed differences in the number of genes differentially expressed might be explained by several factors including OD_600_ of treatment (0.4 vs 0.1), time of H_2_O_2_ challenge (20 min vs 12 min), threshold for considering a gene differentially expressed (fold change of ≥ 2 vs ≥ 3), and different *S*. Typhimurium strains used in the studies (14028s vs 4/74).

### Role of ArcA during aerobic conditions

**Figure 1:**
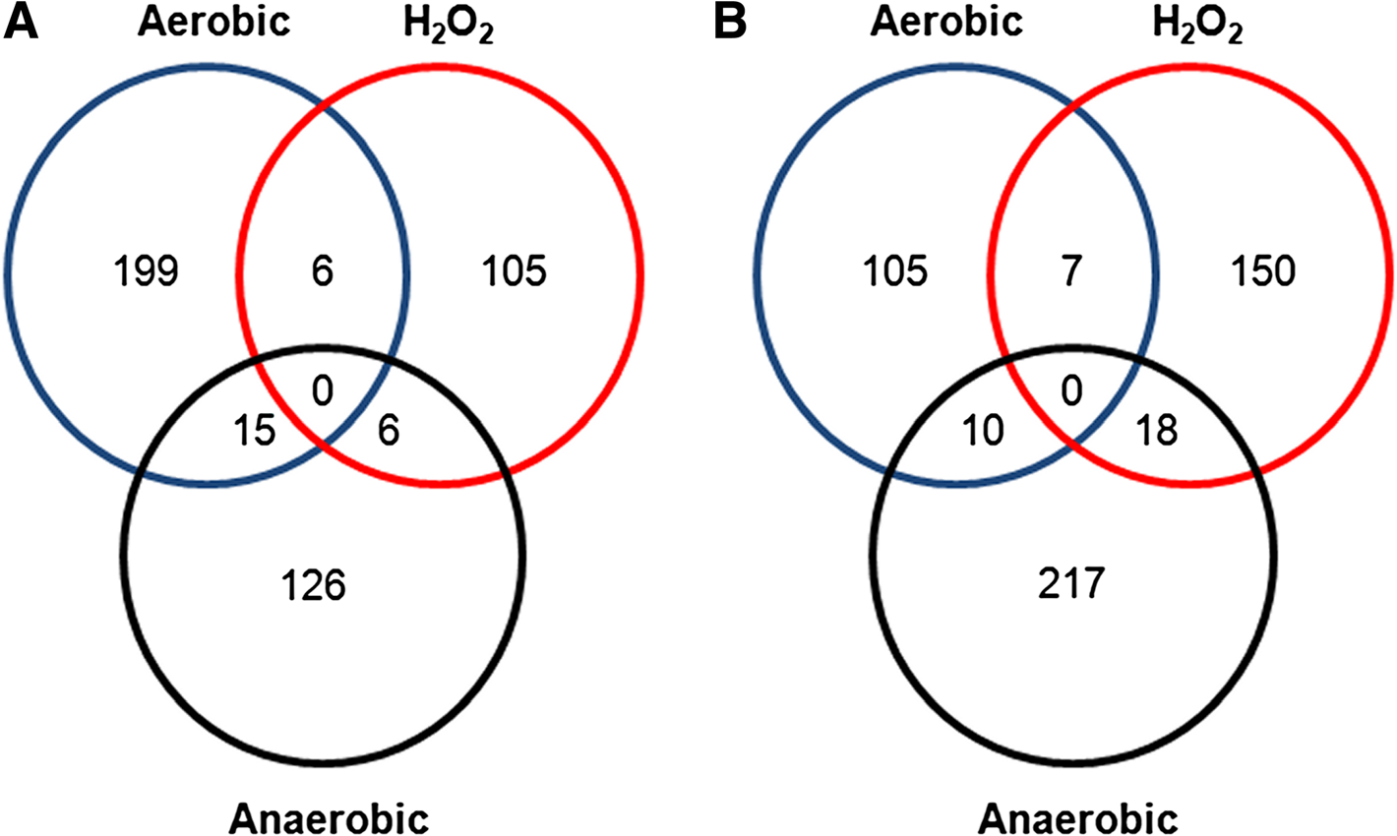
**Overlap of the ArcA regulon in response to H_2_O_2_, aerobiosis and anaerobiosis.** Numbers indicate the amount of genes that show statistically significant differential expression in a Δ*arcA* mutant vs a wild type strain 14028s in the respective condition**.** The number of genes **(A)** positively or **(B)** negatively regulated by ArcA in each condition is shown. Genes regulated by ArcA in anaerobiosis were obtained from [8]. Genes regulated by ArcA in aerobiosis with or without H_2_O_2_ are detailed in Additional file 2: Table S2. Genes with a fold change of ≥ 2 and an FDR q value of ≤ 0.05 for each category were considered to be differentially expressed.

The expression of 220 and 122 genes was up- or downregulated, respectively, by ArcA under aerobic conditions (Additional file 2: Table S2). Comparison with the ArcA regulon of strain 14028s in anaerobiosis [8] showed that 63 genes were regulated under both conditions, but the expression of 38 genes was regulated in the opposite direction. Of the 220 genes upregulated by ArcA in aerobiosis, only 15 are positively regulated by ArcA under anaerobic conditions (Figure 1A), while of the 122 genes downregulated by ArcA under aerobic conditions, only 10 are also downregulated by ArcA in anaerobiosis (Figure 1B).

**Figure 2:**
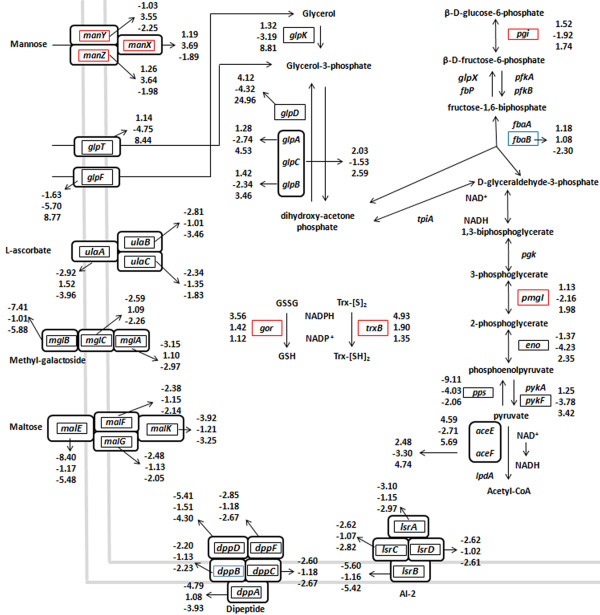
**Effect of ArcA on the expression of genes of central metabolism, sugar uptake and reductive pathways in aerobiosis with or without H_2_O_2_.** Selected genes involved in glycolysis, glycerolipid-, GSH- and thioredoxin- (Trx-[SH]_2_) metabolism, ABC transport and PTS are depicted. Statistically significant differences between strains 14028s and Δ*arcA* in aerobiosis with or without H_2_O_2_ are shown (Table S2). Fold changes are depicted for the aerobically grown wild type strain after H_2_O_2_ treatment (upper value), Δ*arcA* strain after H_2_O_2_ treatment (middle value), and aerobically grown untreated Δ*arcA*/14028s (lower value). Boxes indicate genes regulated by ArcA (Fold change ≥ 2, FDR q-value ≤ 0.05) in aerobiosis (blue), aerobiosis with H_2_O_2_ (red) and under both conditions (black).

To deduce the biological pathways altered in the *arcA* mutant as compared to the aerobically grown wild type strain under aerobic conditions, a Gene Set Enrichment Analysis (GSEA) was performed using the KEGG database for *S*. Typhimurium LT2 as a reference. It should be noted that to determine the pathways regulated by ArcA under aerobic conditions, we compared the transcript levels of the *arcA* mutant to those in the wild type strain (log_2_ Δ*arcA* aerobic – log_2_ wild type aerobic). Thus, pathways repressed by ArcA possess positive NES values (i.e., > 0, glycolysis), while pathways activated by ArcA have negative NES values (i.e., < 0, ABC transporters). GSEA showed that 12 and 8 pathways were up- or downregulated, respectively, by ArcA under aerobic conditions (Table 1). Among the pathways upregulated by ArcA were those implicated in the transport of amino acids and sugars, oligopeptides and metals, including PTS and ABC transporters, among others (Table 1, negative NES values). Under aerobic conditions, ArcA mainly repressed pathways implicated in central metabolism and nucleotide biosynthesis (Table 1, positive NES values). In particular, the transcript levels of genes encoding proteins of the payoff phase of glycolysis [42] and glycerolipid metabolism were higher in the *arcA* mutant grown under aerobic conditions than in strain 14028s (Figure 2, Addition file 2 Table S2), including *pykF* (pyruvate kinase), *aceEF*-*lpdA* (pyruvate dehydrogenase complex), *eno* (enolase), *glpD* and *glpABC* (glycerol 3-phosphate dehydrogenase). The data suggests that the aerobically grown Δ*arcA* mutant presents a higher flux through glycolysis and increased levels of NADH than the wild type strain. Interestingly, genes of the Krebs cycle, which are negatively regulated by ArcA under anaerobic conditions [8], were not repressed in aerobiosis (Table S2). Together, these results indicate that ArcA has a major role in regulating gene expression under aerobic conditions and that the genes regulated in aerobiosis are different from those regulated in anaerobiosis.

**Table 1:** Pathways differentially regulated (q ≤ 0.25) by ArcA in response to H_2_O_2_ and aerobiosis as determined by GSEA

		**Genes regulated**		**FDR q-value**		**NES**^**H**^	
**Gene set**	**Size of gene set**^**A**^	**Aerobic**^**B**^	**H**_**2**_**O**_**2**_^**C**^	**Aerobic**^**D**^	**H**_**2**_**O**_**2**_^**E**^	**Aerobic**^**F**^	**H**_**2**_**O**_**2**_^**G**^
Glycerophospholipid metabolism	25	5	5	0.001	0.000	2.16	2.29
Purine metabolism	73	19	35	0.003	0.000	2.06	2.22
Pyrimidine metabolism	50	14	18	0.044	0.003	1.79	2.02
Lipopolisaccharide biosynthesis	26		17		0.004		1.98
Glutathione metabolism	16		8		0.005		1.94
Bacterial invasion of epithelial cells	8	8	5	0.002	0.012	−1.9	1.87
Glycolysis/gluconeogenesis	33	10	10	0.250	0.022	0.46	1.81
Biosynthesis of siderophore group non-ribosomal peptides	5		4		0.067		1.69
Glycerolipid metabolism	10	1	1	0.083	0.109	1.68	1.6
Flagellar assembly	34		7		0.189		1.5
Phosphotransferase system (PTS)	32	17	17	0.001	0.000	−1.88	−2.26
ABC transporters	124	46	29	0.005	0.022	−1.83	−1.86
*Salmonella* infection	16	8		0.001		−2.04	
Bacterial secretion system	28	12		0.005		−1.79	
Bacterial chemotaxis	22	6		0.049		−1.63	
Two-component system	88	11		0.096		−1.56	
Cyanoamino acid metabolism	6	3		0.129		−1.52	
Ascorbate and aldarate metabolism	8	3		0.117		−1.52	
Amino sugar and nucleotide sugar metabolism	50	14		0.213		−1.43	
Fructose and mannose metabolism	34	14		0.233		−1.43	
Starch and sucrose metabolism	26	14		0.244		−1.39	
Ribosome	45	28		0.004		2.01	
Riboflavin metabolism	8	3		0.113		1.63	
Cysteine and methionine metabolism	24	8		0.137		1.59	

^A^Based on the KEGG database for *S*. Typhimurium LT2.

^B^Number of genes predicted to contribute to the enrichment in aerobic growth in rich media.

^C^Number of genes predicted to contribute to the enrichment in response to H_2_O_2_.

^D^q-value for the pathway predicted to be regulated by ArcA in aerobic growth in rich media.

^E^q-value for the pathway predicted to be regulated by ArcA in response to H_2_O_2_.

^F^Normalized enrichment score for pathways under aerobic conditions. Positive values indicate pathways negatively regulated by ArcA, while negative values indicate pathways that are up-regulated by ArcA.

^G^Normalized enrichment score for pathways under aerobic conditions after H_2_O_2_ exposure. Positive values indicate pathways positively regulated by ArcA, while negative ones indicate pathways that are downregulated by ArcA.

^H^Normalized enrichment score.

### Role of ArcA in the response to H_2_O_2_ under aerobic conditions

It has been well established that an aerobically grown Δ*arcA* strain is sensitive to H_2_O_2_ treatment [7-10], however, the genes regulated by ArcA under this condition have not been determined. Our analysis shows that ArcA directly or indirectly regulates the expression of 292 genes in response to H_2_O_2_ in aerobically grown cells, 117 and 175 genes were up- or downregulated, respectively (Figure 1, Additional file 2: Table S2). Surprisingly, almost no correlation was observed between the genes regulated by ArcA in aerobiosis with or without H_2_O_2_: only 6 genes were upregulated under both conditions (Figure 1A), while 7 genes were downregulated (Figure 1B). Furthermore, comparison of the ArcA regulon in aerobiosis, anaerobiosis [8] and aerobiosis with H_2_O_2_ showed that no genes were up or downregulated in all three conditions (Figure 1A and B). This suggests that the genes regulated by ArcA in response to various stimuli are different and do hardly overlap (Figure 1), which underscores the importance and versatility of ArcA-mediated regulation.

To determine the pathways regulated by ArcA in response to H_2_O_2_ under aerobic conditions, the intensity values were treated as follows: (log_2_ 14028s wild type H_2_O_2_ aerobic - log_2_ 14028s wild type aerobic) - (log_2_ Δ*arcA* H_2_O_2_ aerobic - log_2_ Δ*arcA* aerobic). Therefore, in contrast to the pathways regulated by ArcA under aerobic conditions, the pathways positively regulated by ArcA in response to H_2_O_2_ under aerobic conditions have positive NES values, while the pathways negatively regulated by ArcA in response to H_2_O_2_ under aerobic conditions have negative NES values. Based on the transcriptomic data (Additional file 2: Table S2), GSEA deduced that 10 and 2 pathways were up- or downregulated, respectively, by ArcA in aerobically grown cells after H_2_O_2_ exposure (Table 1). The pathways deduced to be upregulated by ArcA in response to H_2_O_2_ are implicated in nucleotide and siderophore biosynthesis, central and glutathione metabolism, among others, while the pathways downregulated by ArcA were PTS and ABC transporters (Figure 2, Table 1). Interestingly, only one gene (*ahpF*) required for H_2_O_2_ degradation was upregulated by ArcA in aerobiosis after H_2_O_2_ treatment (Additional file 2: Table S2), suggesting that ArcA is not required for ROS scavenging. Of particular interest are the genes most upregulated by ArcA in aerobiosis with H_2_O_2_ in the pathways of nucleotide and glutathione metabolism, coding for the alternative ribonucleotide reductase (*nrdEF*) and glutathione reductase (*gor*). In addition, the gene coding for thioredoxin reductase (*trxB*), required for reduction of oxidized thioredoxin (Trx-[S]_2_), was also upregulated by ArcA after peroxide exposure. In *E. coli*, *nrdEF* is upregulated in response to H_2_O_2_[43,44] and allows replication in iron-limiting conditions when manganese is present [44]. Gor and TrxB are required for the turnover of oxidized glutathione (GSSG) and Trx-[S]_2_, respectively (reduction from GSSG to reduced glutathione (GSH), and Trx-[S]_2_ to reduced thioredoxin (Trx-[SH]_2_), respectively), which participate in the reduction of disulfide bonds [45]. This suggests that in aerobically grown cells exposed to H_2_O_2_, ArcA regulates the GSH/GSSG and Trx-[S]_2_/Trx-[SH]_2_ ratio, modulating the redox status of the cell, and the expression of *nrdEF*.

### Promoter analysis

To determine the genes that may be directly regulated by ArcA, we identified the subset of ArcA-dependently differentially expressed genes in aerobiosis with or without H_2_O_2_ with predicted ArcA-binding sites in the upstream regions (Additional file 2: Table S2), using the sequenced genome of *S*. Typhimurium 14028s [37] and Matrix-scan [38], as detailed in Methods. The analysis predicted that ArcA directly regulates the expression of 6 genes in aerobiosis with H_2_O_2_ and 19 genes in aerobically grown cells (Table 2).

**Table 2:** Genes differentially expressed by ArcA under aerobic conditions with or without H_2_O_2_ that have predicted ArcA binding sites

**Gene ID**	**Gene ID**	**Gene name**	**Fold change H**_**2**_**O**_**2**_**/control**		**Fold change control**	**Strand**	**Position**^**A**^	**Sequence**	**p-value**^**B**^	**Function**
**LT2**	**14028s**		**14028s**	Δ***arcA***	Δ***arcA*/14028s**			**5′ - 3′**		
STM0958	STM14_1080.J	*trxB*	4.93	1.9	1.35	-	94	GTTAACAATATGTGT	1.00E-05	thioredoxin reductase
						+	85	GTTAACAAAATCGTT	5.70E-05	
STM1520	STM14_1838	*marR*	1.75	−1.38	−1.02	-	73	GTCAACTAAATGAAT	9.50E-05	DNA-binding transcriptional repressor MarR
STM1586	STM14_1918	*-*	5.55	2.75	1.06	+	171	GTTAAGAAAATGTGC	9.50E-05	putative periplasmic protein
STM3216	STM14_3893	*tsr*	1.17	−1.65	−1.31	-	198	GTTAACCATTTCTTA	8.10E-06	putative methyl-accepting chemotaxis protein
STM2445	STM14_3003	*ucpA*	−5.34	−1.83	−1.86	+	44	GTTAATGGAGTGTAA	1.20E-05	short chain dehydrogenase
STM1795	STM14_2170	*gluD*	−6.28	−1.34	−1.94	-	121	GTTAACTATCCGCTA	9.50E-05	putative glutamic dehyrogenase-like protein
STM4087	STM14_4913	*glpF*	−1.63	−5.7	8.77	+	217	GTTAATGAAATGATT	1.00E-05	glycerol diffusion
STM1771	STM14_2141	*chaA*	−1.77	−5.97	3.31	-	36	GTTAATATTTTGGAA	8.00E-05	calcium/sodium:proton antiporter
STM1125	STM14_1281	*putP*	−8.81	−3.4	−3.46	+	234	GTTAACACTTTTAAA	9.50E-05	major sodium/proline symporter
STM1091	STM14_1237	*sopB*	−2.17	−3.57	−5.89	+	52	GTTAACCCTGTTGAA	8.00E-05	secreted effector protein
STM2866	STM14_3463	*sprB*	−6.06	−2.32	−3.51	+	281	GTTAATGAAAGGGAA	8.10E-06	transcriptional regulator
STM4405	STM14_5290	*ytfJ*	−3.2	−1.45	−2.31	-	67	GTTAATCATATGTGC	3.30E-05	putative transcriptional regulator
STM4535	STM14_5449	*-*	−2.7	−1.04	−2.76	-	98	GTTAACAGAGGGAAA	9.50E-05	putative PTS permease
STM4467	STM14_5361		−1.83	1.02	−2.07	-	271	GTTAATTATTTGTTT	6.50E-06	arginine deiminase
STM1130	STM14_1293	*nanM*	−2.58	1.01	−2.92	+	115	GATAACTCCATGTAA	8.00E-05	putative inner membrane protein
STM4165	STM14_5006	*rsd*	2.75	1.69	2.74	-	67	GTTAACAACATGCCA	1.20E-05	anti-RNA polymerase sigma 70 factor
STM1728	STM14_2091	*yciG*	1.68	1.95	−2.11	-	261	GTTAATGCATTGTTT	1.50E-05	putative cytoplasmic protein
STM0292	STM14_0341	*-*	1.18	2.12	2.18	-	248	GTTCATCAAATGTAG	6.80E-05	putative RHS-like protein
STM2220	STM14_2744	*yejG*	5.84	2.41	2.94	+	64	GTCAATGATGTGTTA	6.80E-05	hypothetical protein
STM1770	STM14_2140	*chaB*	2.07	3.64	−2.12	+	245	GTTAATATTTTGGAA	8.00E-05	cation transport regulator
STM1211	STM14_1385.J	*ndh*	7.09	3.58	2.24	-	44	GTTAATTAAAAGTTA	1.10E-06	respiratory NADH dehydrogenase 2
						+	65	GTTAATTAAAGGCTA	1.00E-05	
						-	33	ATTAACCAATTGTTA	9.50E-05	
STM1746.S	STM14_2110	*oppA*	−7.87	−2.46	−3.08	+	318	GTTAACAAAATTGTA	1.00E-05	oligopeptide transport protein
						-	327	GTTAACCAATTCTCT	6.80E-05	
STM1818	STM14_2199	*fadD*	−1.56	−1.85	2.56	+	75	GTTAATATAATGTTA	1.00E-05	long-chain-fatty-acid--CoA ligase
						+	64	GTTAACGACTTGTTT	1.00E-05	
STM3692	STM14_4451	*lldP*	−13.27	−2.11	−6.83	-	125	GTTAACCAGATGTTA	2.00E-06	L-lactate permease
						+	136	GTTAACTATTTGTTG	5.20E-06	
						-	173	GTTAATTTAATGAAA	1.90E-05	
STM1303	STM14_1582	*argD*	−2.63	−1.11	−2.95	-	40	GTTATTTATATGTTA	2.80E-05	bifunctional succinylornithine transaminase
						+	112	GTTTATGCAATGTTA	5.70E-05	

^A^location of the binding sequence is in bp upstream of the translation start site in the genome of *S*. Typhimurium 14028s [37].

^B^p-value estimates the significance of the weight associated to each site [38].

### Biochemical analysis of the Δ*arcA* strain

The microarray analysis predicted that in response to H_2_O_2_ under aerobic conditions, ArcA regulates the expression of genes implicated in GSH metabolism. It also predicted that in aerobiosis, ArcA regulates expression of genes coding proteins involved in glycolysis (Table 1 and Additional file 2: Table S2). To evaluate if the changes in gene expression correlated with changes in the products of these pathways, we determined the levels of GSH, glutathione reductase (GR) activity, NADH and total intracellular ROS in the wild type and Δ*arcA* strains in aerobiosis with and without H_2_O_2_.

**Figure 3:**
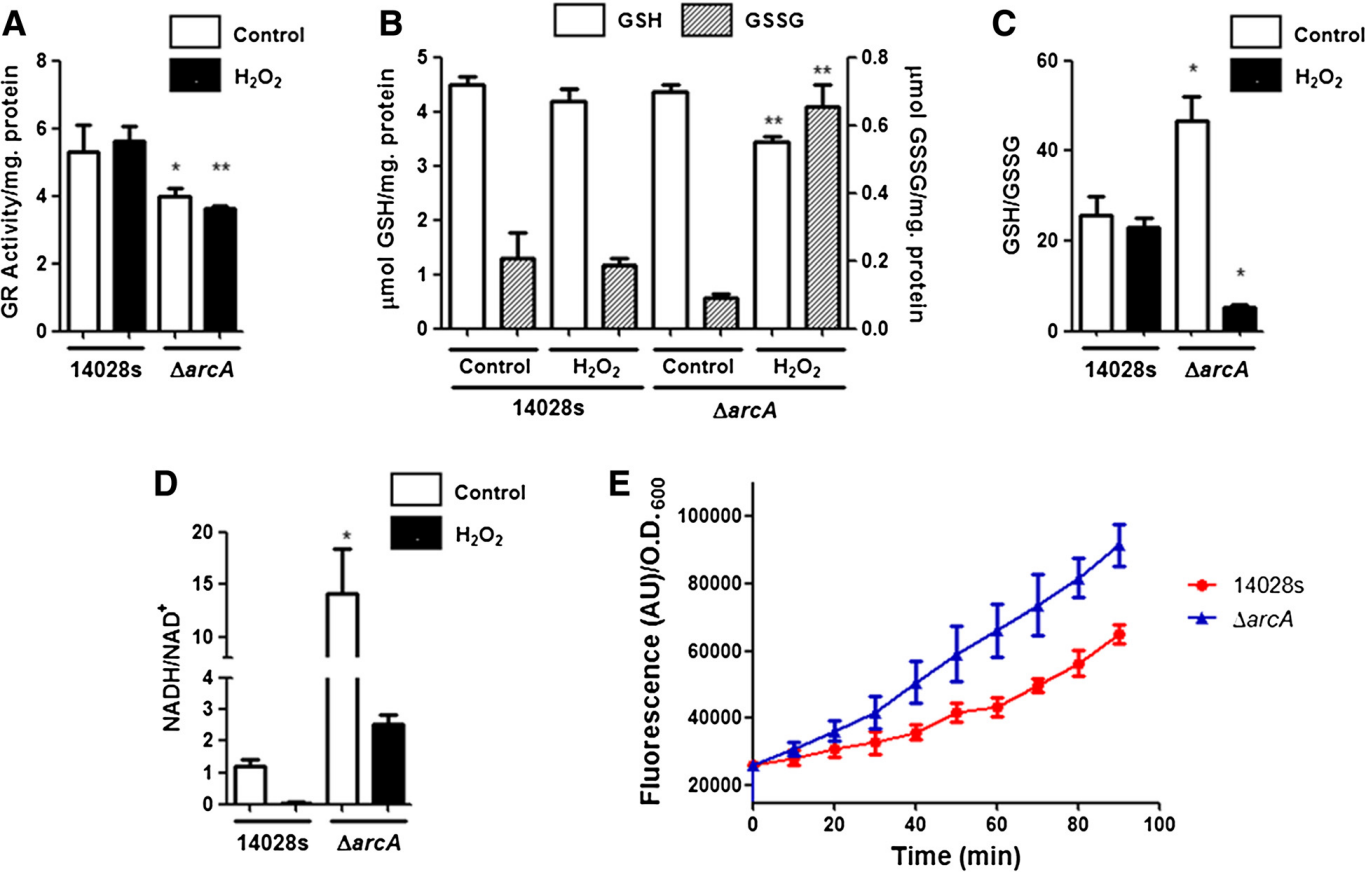
**Role of ArcA in modulating the redox status in aerobiosis and in response to H_2_O_2_.** Strains 14028s and Δ*arcA* were grown to OD_600_ ~ 0.4 and treated with 1.0 mM H_2_O_2_ for 20 min. Control cells received no treatment. The levels of **(A)** glutathione reductase (GR) activity, **(B)** reduced glutathione (GSH), oxidized glutathione (GSSG), **(C)** the GSH/GSSG ratio and **(D)** the NADH/NAD^+^ ratio were measured. **(E)** Total ROS amount was measured under aerobic conditions. Values are the mean ± SD of three independent experiments. Values were normalized by protein concentration (A, B, C, D) or OD_600_**(E)**. AU: arbitrary units. GR activity: nmol NADPH min^-1^ ml^-1^ mg protein^-1^. *p ≤ 0.05, **p ≤ 0.01, as compared to aerobically grown wild type cells.

The gene *gor* was upregulated by ArcA under aerobic conditions with H_2_O_2_ (Figure 2, Additional file 2: Table S2), therefore decreased levels of both GSH and GR activity in the *arcA* mutant exposed to H_2_O_2_ under aerobic conditions were expected. The levels of GR activity were indeed lower in the aerobically grown Δ*arcA* strain after H_2_O_2_ treatment, although the levels of GR activity were also decreased in the Δ*arcA* mutant under aerobic conditions (Figure 3A). GSH remained almost unaltered in the wild type strain after treatment with the toxic compound, conversely, the aerobically grown Δ*arcA* mutant treated with H_2_O_2_ showed significantly reduced levels of GSH and increased GSSG, consistent with lower GR activity (Figure 3A and B). In agreement, the GSH/GSSG ratio was lower in the aerobically grown Δ*arcA* strain after H_2_O_2_ treatment (Figure 3C), while the levels of total glutathione were similar between the wild type and Δ*arcA* mutant strains (4.68 and 4.45 μmol/mg. protein, respectively). This indicates that GSH turnover is altered in an aerobically grown Δ*arcA* strain with H_2_O_2_ due to lower GR activity.

The transcript levels of genes coding the pyruvate dehydrogenase complex (PDH), proteins of the payoff phase of glycolysis [42] and sugar uptake were higher in the aerobically grown Δ*arcA* mutant than in the wild type strain (Figure 2, Additional file 2: Table S2). This suggests that under aerobic conditions a Δ*arcA* strain has a higher flux through glycolysis, which in turn could result in higher levels of acetyl-CoA and an elevated NADH generation in the Krebs cycle. As predicted, the NADH/NAD^+^ ratio was significantly lowered in the aerobically grown wild type strain after peroxide treatment (Figure 3D), compared to untreated aerobically grown wild type cells. In the Δ*arcA* mutant, the NADH/NAD^+^ ratio was higher than in the wild type strain in aerobically grown cells before and after H_2_O_2_ treatment (Figure 3D). Although there was an overall decrease in the NADH/NAD^+^ ratio in the Δ*arcA* strain after H_2_O_2_ treatment, the ratio remained 2-fold higher than in wild type cells under aerobic conditions without H_2_O_2_ treatment.

In *E. coli,* one of the sources of O_2_^-^ is oxidation of the respiratory electron transport chain and the conversion of NADH to NAD^+^[46]. Since under aerobic growth conditions a Δ*arcA* strain has higher levels of NADH (Figure 3D) and *ndh* transcript than the wild type strain (Additional file 2: Table S2), we hypothesized that a Δ*arcA* mutant might present increased levels of total ROS. In agreement, in an aerobically grown Δ*arcA* strain, total ROS was increased as compared to the isogenic wild type strain under the same condition (Figure 3E), indicating that the absence of ArcA generates a metabolic imbalance which leads to increased levels of ROS.

In order to complement the Δ*arcA* mutation, we first evaluated the mechanism by which ArcA regulates gene expression in response to ROS. Our results show that in *S*. Typhimurium 14028s, *arcA* expression is not increased either with H_2_O_2_ or hypochlorous acid (Additional file 1: Figure S1A). In addition, the levels of ArcA also remained constant after exposure to both ROS (Additional file 1: Figure S1B). This suggests that rather than changes in expression, ArcA is activated in response to ROS, most likely by phosphorylation of residue D54. To test this hypothesis, the Δ*arcA* mutant strain was complemented in *trans* with the wild type gene and a version coding a substitution of residue D54 of ArcA (D54A), and the number of colony forming units (CFU/ml) was determined after H_2_O_2_ exposure. As predicted, only complementation with the wild type gene resulted in similar CFU/ml as in strain 14028s (Additional file 1: Figure S1C), however, there were also differences in the number of CFU/ml at the initial time points. This is most likely caused by increased levels of ArcA due to complementation with a high copy number vector. Since the effect of ArcA is pleiotropic and its levels remain constant throughout all stresses evaluated (Additional file 1: Figure S1 A and B), achieving wild type levels of ArcA is required to properly address its role in the response to ROS.

## Discussion

**Figure 4:**
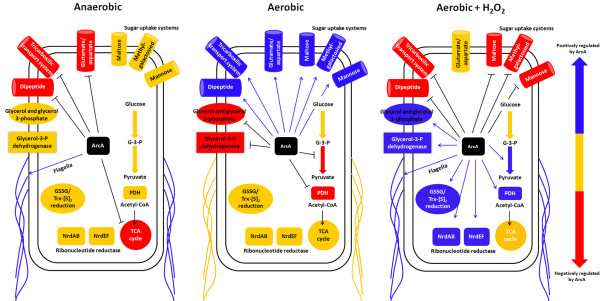
**Model showing the major differences in the processes regulated by ArcA after H_2_O_2_ exposure under aerobic conditions, aerobiosis and anaerobiosis in *S. *Typhimurium 14028s.** Each symbol represents groups of genes that are functionally related and are colored based on the degree of regulation by ArcA under each condition. Data from anaerobic conditions was obtained from [8]. Blue shows that the genes are positively regulated by ArcA, yellow shows that the genes are not affected by ArcA, and red shows that the genes are negatively regulated by ArcA. G-3-P: glyceraldehyde 3 phosphate, PDH: pyruvate dehydrogenase complex, TCA cycle: tricarboxylic acid cycle, GSSG: oxidized glutathione, Trx-[S]_2_: oxidized thioredoxin.

Several reports have demonstrated that the global regulator ArcA is required for H_2_O_2_ resistance [7-10], however, only a few have evaluated its role on regulating gene expression under this condition [27,28]. One study conducted in *E. coli* used a proteomic approach to evaluate the mechanism underlying the role of ArcA in response to ROS [7]. Herein, we report the first genome-wide study addressing the role of ArcA in response to H_2_O_2_ under aerobic conditions. ArcA regulates different genes after ROS exposure in aerobiosis, under aerobic growth in rich media and under anaerobiosis (Figure 1, 2 and 4; Additional file 2: Table S2). In this discussion, we will focus on the genes and pathways regulated by ArcA that contribute to ROS resistance of *S*. Typhimurium. A full list of the genes regulated by ArcA in aerobiosis with and without H_2_O_2_ is provided in Additional file 2: Table S2.

### Role of ArcA in ROS scavenging

The *S*. Typhimurium genome codes for several genes that degrade H_2_O_2_ or organic hydroperoxide, including catalases (*katG*, *katE* and *katN*), alkyl hydroperoxide reductases (*ahpCF* and *tsaA*) [47] and a glutathione-dependent peroxidase (*btuE*) [48]. Only *ahpF* and *katE* are predicted to be regulated by ArcA in aerobiosis with or without H_2_O_2_, respectively (Additional file 2: Table S2). Expression of *ahpF* and *katE* is also known to be regulated by RpoS [49,50]. Neither *katN* nor *sodA*, previously described as members of the ArcA regulon under anaerobic conditions [8,51], were found to be regulated by ArcA under aerobic conditions with or without H_2_O_2_ treatment. These results are in agreement with studies in *E. coli* that demonstrate that an *arcA* mutant does not show defects in H_2_O_2_ scavenging [7].

### Role of ArcA in maintaining GSH and thioredoxin levels

ArcA positively regulates the expression of the genes *gor* (GR) and *trxB* (thioredoxin reductase) in aerobiosis with H_2_O_2_, but not without the toxic compound (Figure 2 and 4 Additional file 2: Table S2). However, the levels of GR activity were lower in the *arcA* mutant strain grown under aerobic conditions (Figure 3A), but not the levels of GSH turnover (Figure 3B). This suggests that there are other unidentified factors that alter GR activity in the *arcA* mutant grown under aerobic conditions, since there are no differences in the transcript levels of the gene *gor* between strains 14028s and Δ*arcA* (Additional file 2: Table S2). In addition, this indicates that the lower levels of GR activity in the *arcA* mutant grown under aerobic conditions are sufficient to cope with GSH turnover, and the effect is only evident when GSH oxidation is increased, as when cells are exposed to peroxide.

In *E. coli*, OxyR regulates *gor* expression [6], while the expression of *trxB* has not been determined under this condition. Two putative ArcA binding sites were predicted at the promoter region of *trxB* (Table 2). Glutathione and thioredoxin reductases are required to reduce GSSG and thioredoxin in a NADPH-dependant manner, which in their reduced form participate in the reduction of cellular disulfide bonds [45] and of oxidized glutaredoxin. An *E. coli* Δ*gor* mutant has a slight sensitivity towards paraquat and cumene hydroperoxide [52], while in stationary phase a Δ*trxB* strain shows H_2_O_2_ sensitivity [53]. However, a double Δ*gor* Δ*trxB* mutant grows extremely poorly under aerobic conditions and presents increased alkaline phosphatase activity, indicative of increased disulfide bond formation, most likely due to increased ROS [54]. Since an aerobically grown Δ*arcA* mutant treated with H_2_O_2_ has lower transcript levels of *gor* and *trxB*, lower GR activity and lower GSH levels (Figure 2 and 3A, B and C), this might result in increased disulfide bond formation, protein inactivation and contribute to the increased sensitivity towards ROS. Since GSH is abundant in the cell and is readily oxidized by H_2_O_2_, this leads to a decrease in the levels of reduced glutathione, shifting the target of oxidation from GSH to essential macromolecules, leading to cell death [55]. This may occur earlier in a Δ*arcA* mutant, as its level of “protective” GSH is low, caused by decreased GR activity.

### ArcA and nucleotide metabolism

The pathways that showed the highest changes in the Δ*arcA* mutant treated with H_2_O_2_ under aerobic conditions were purine and pyrimidine metabolism (Figure 4, Table 1). The major differences are found in the expression of the *nrdAB* and *nrdEFHI* operons, coding for aerobic and alternative aerobic ribonucleotide reductase, respectively. In *E. coli* and *S*. Typhimurium, NrdAB is indispensable for growth under aerobic conditions while NrdEF is not functional [56]. In the aerobically grown wild type strain, *nrdAB* was repressed while *nrdEF* was up-regulated after H_2_O_2_ exposure, in agreement with studies in *E. coli* and *S*. Typhimurium 4/74 [41,43], while the regulation was lost in the Δ*arcA* mutant under the same conditions (Additional file 2: Table S2). NrdEF is usually repressed by Fur. However, in response to H_2_O_2,_ this Fur repression is abolished and the apoprotein form of IscR upregulates expression of the operon [44]. This suggests that in response to H_2_O_2_ under aerobic conditions, ArcA may act together with Apo-IscR, up-regulating the *nrdEFHI* operon.

### ArcA and carbon metabolism

Under aerobic conditions, the transcript levels of genes coding proteins of glycerolipid metabolism, glycolysis and the PDH complex were higher in the Δ*arcA* mutant than in the wild type strain (Figure 2 and 4, Additional file 2: Table S2). This suggests that the flux through glycolysis and the levels of acetyl-CoA could be increased in the Δ*arcA* strain. Two studies conducted in *E. coli* measured the flux through the PDH complex in a Δ*arcA* mutant under aerobic conditions with different results. One showed that there was an increase in the flux through the PDH complex [14] while in the other no differences were observed [57], although both studies determined that there was an increase in the flux through the TCA cycle. Our analysis showed that the NADH/NAD^+^ ratio was 2-fold higher in the aerobically grown Δ*arcA* mutant than in the wild type strain (Figure 3D). After H_2_O_2_ exposure, the NADH/NAD^+^ ratio decreased in the wild type and Δ*arcA* strain, but in the latter the levels remained higher than in the wild type strain under aerobic conditions (Figure 3D). Since NADH can reduce Fe^3+^ to Fe^2+^*in vitro*[56], and elevated NADH levels result in increased sensitivity towards H_2_O_2_[58], the higher basal levels of NADH in the Δ*arcA* mutant in aerobiosis and after H_2_O_2_ treatment may increase Fe^+2^ turnover, fueling the Fenton reaction (the formation of OH·, and Fe^3+^ from the nonenzymatic reaction of Fe^2+^ with H_2_O_2_) and leading to higher levels of ROS-derived damage.

In the respiratory chain, NADH dehydrogenase II (encoded by *ndh*) generates O_2_^-^ and H_2_O_2_ by oxidation of its reduced FADH_2_ cofactor [58]. In an aerobically grown Δ*arcA* strain, the levels of NADH and the *ndh* transcript (Additional file 2: Table S2) are higher than in the wild type strain under the same condition (Figure 3D). We therefore speculated that production of intracellular ROS might be increased. In agreement, a Δ*arcA* mutant presents statistically significant increased levels of total ROS as compared to the wild type strain 14028s (Figure 3E). These higher levels of ROS might present further disadvantages for the bacterium when exposed to H_2_O_2_. However, several other sources of intracellular ROS besides NADH dehydrogenase II may also contribute to the higher levels of ROS observed in the Δ*arcA* mutant, such as fumarate-reducing flavoenzymes [59].

## Conclusion

We identified the ArcA regulon in *S*. Typhimurium under aerobic growth with and without H_2_O_2_, and show that ArcA coordinates a response that includes changes in cellular-, glutathione-, thioredoxin-, NADH- and glycerolipid metabolism. These changes contribute to H_2_O_2_ resistance by modulating the reducing potential of the cell.

## Competing interests

The author(s) declare that they have no competing interests.

## Author’s contributions

EHM and CPS conceived the project. EHM and PD conducted the analysis of microarray data and prediction of regulated pathways. EHM, BC and ILC performed the experiments. FG, RL and SP conducted partial data analysis. EHM, SP, MM and CPS wrote the paper. All authors read and approved the final manuscript. The authors declare no conflict of interest.

## Supplementary Material

Additional file 1 **Probing the ArcA regulon under aerobic/ROS conditions in *Salmonella enterica *serovar Typhimurium. A)** Supplementary methods. **B)** Figure S1: Characterization of the mechanism of ArcA in response to ROS. Measurement of the transcript and protein levels of *arcA* by qRT-PCR and Western blot, respectively. Determination of CFU/ml in strains 14028s, Δ*arcA*, Δ*arcA*::*cat*/pBR::*arcA*, and Δ*arcA*::*cat*/pBR::*arcA*D54A, after H_2_O_2_ exposure. **C)** Table S1: Validation of microarray data using qRT-PCR of randomly selected genes. Fold changes are given for the selected genes in response to hydrogen peroxide in the different genetic backgrounds as determined by qRT-PCR and microarray analysis. **D)** Supplementary references [60]. Click here for file

Additional file 2: Table S2 Table of genes that showed intensity values over the background. Fold changes are given for every gene in response to H_2_O_2_ in the different genetic backgrounds. Click here for file

## References

[B1] Mackaness GB Blanden RV Collins FM Host-parasite relations in mouse typhoid J Exp Med 1966 124 573 583 10.1084/jem.124.4.573 5922285 PMC2138243

[B2] Fridovich I The biology of oxygen radicals Science 1978 201 875 880 10.1126/science.210504 210504

[B3] Hassett D Cohen M Bacterial adaptation to oxidative stress: implications for pathogenesis and interaction with phagocytic cells FASEB J 1989 3 2574 2582 2556311 10.1096/fasebj.3.14.2556311

[B4] Imlay J Pathways of Oxidative Damage Annu Rev Microbiol 2003 57 395 418 10.1146/annurev.micro.57.030502.090938 14527285

[B5] Canvin J Langford PR Wilks KE Kroll JS Identification of sodC encoding periplasmic [CuZn]-superoxide dismutase in Salmonella FEMS Microbiol Lett 1996 136 215 220 10.1111/j.1574-6968.1996.tb08052.x 8869506

[B6] Christman MF Morgan RW Jacobson FS Ames BN Positive control of a regulon for defenses against oxidative stress and some heat-shock proteins in *Salmonella typhimurium* Cell 1985 41 753 762 10.1016/S0092-8674(85)80056-8 2988786

[B7] Loui C Chang AC Lu S Role of the ArcAB two-component system in the resistance of *Escherichia coli* to reactive oxygen stress BMC Microbiol 2009 9 183 10.1186/1471-2180-9-183 19715602 PMC2748088

[B8] Evans MR Fink RC Vazquez-Torres A Porwollik S Jones-Carson J McClelland M Hassan HM Analysis of the ArcA regulon in anaerobically grown *Salmonella enterica* sv Typhimurium. BMC Microbiol 2011 21 11 58 10.1186/1471-2180-11-58 PMC3075218 21418628

[B9] Lu S Killoran PB Fang FC Riley LW The global regulator ArcA controls resistance to reactive nitrogen and oxygen intermediates in *Salmonella enterica* serovar Enteritidis Infect Immun 2002 70 451 461 10.1128/IAI.70.2.451-461.2002 11796570 PMC127680

[B10] Wong SM Alugupalli KR Ram S Akerley BJ The ArcA regulon and oxidative stress resistance in *Haemophilus influenzae* Mol Microbiol 2007 64 1375 1390 10.1111/j.1365-2958.2007.05747.x 17542927 PMC1974803

[B11] Iuchi S Matsuda Z Fujiwara T Lin EC The *arcB* gene of *Escherichia coli* encodes a sensor-regulator protein for anaerobic repression of the arc modulon Mol Microbiol 1990 4 715 727 10.1111/j.1365-2958.1990.tb00642.x 2201868

[B12] Iuchi S Lin EC Purification and phosphorylation of the Arc regulatory components of *Escherichia coli* J Bacteriol 1992 174 5617 5623 1512197 10.1128/jb.174.17.5617-5623.1992 PMC206507

[B13] Rolfe MD Ter Beek A Graham AI Trotter EW Shahzad Asif HM Sanguinetti G de Mattos JT Poole RK Green J SysMO-SUMO Transcript profiling and inference of *Escherichia coli* K-12 ArcA activity across the range of physiologically relevant oxygen concentrations J Biol Chem 2011 286 10147 10154 10.1074/jbc.M110.211144 21252224 PMC3060466

[B14] Alexeeva S Hellingwerf K Mattos JT Requirement of ArcA for Redox Regulation in *Escherichia coli* under Microaerobic but Not Anaerobic or Aerobic Conditions J Bacteriol 2003 185 204 209 10.1128/JB.185.1.204-209.2003 12486057 PMC141817

[B15] Georgellis D Kwon O Lin EC Quinones as the Redox Signal for the Arc Two-Component System of Bacteria Science 2001 292 2314 2316 10.1126/science.1059361 11423658

[B16] Malpica R Franco B Rodriguez C Kwon O Georgellis D Identification of a quinone-sensitive redox switch in the ArcB sensor kinase Proc Natl Acad Sci 2004 101 13318 13323 10.1073/pnas.0403064101 15326287 PMC516565

[B17] Bekker M Alexeeva S Laan W Sawers G Mattos JT Hellingwerf K The ArcBA Two-Component System of *Escherichia coli* Is Regulated by the Redox State of both the Ubiquinone and the Menaquinone Pool J Bacteriol 2010 191 746 754 19933363 10.1128/JB.01156-09 PMC2812447

[B18] Peña-Sandoval G Georgellis D The ArcB Sensor Kinase of *Escherichia coli* Autophosphorylates by an Intramolecular Reaction J Bacteriol 2010 192 1735 1739 10.1128/JB.01401-09 20097862 PMC2832521

[B19] Georgellis D Lynch AS Lin EC In vitro phosphorylation study of the arc two-component signal transduction system of *Escherichia coli* J Bacteriol 1997 179 5429 5435 9286997 10.1128/jb.179.17.5429-5435.1997 PMC179413

[B20] Kwon O Georgellis D Lin EC Phosphorelay as the Sole Physiological Route of Signal Transmission by the Arc Two-Component system of *Escherichia coli* J Bacteriol 2000 182 3858 3862 10.1128/JB.182.13.3858-3862.2000 10851007 PMC94563

[B21] Lynch AS Lin EC Transcriptional control mediated by the ArcA two-component response regulator protein of *Escherichia coli*: characterization of DNA binding at target promoters J Bacteriol 1996 178 6238 6249 8892825 10.1128/jb.178.21.6238-6249.1996 PMC178496

[B22] Jeon Y Lee Y Han J Kim J Hwang D Multimerization of Phosphorylated and Non-phosphorylated ArcA is Necessary for the Response Regulator Function of the Arc Two-Component Signal Transduction System J Biol Chem 2001 276 40873 40879 10.1074/jbc.M104855200 11527965

[B23] Oshima T Aiba H Masuda Y Kanaya S Sugiura M Wanner BL Mori H Mizuno T Transcriptome analysis of all two-component regulatory system mutants of *Escherichia coli* K-12 Mol Microbiol 2002 46 281 291 10.1046/j.1365-2958.2002.03170.x 12366850

[B24] Liu X De Wulf P Probing the ArcA-P modulon of *Escherichia coli* by whole genome transcriptional analysis and sequence recognition profiling J Biol Chem 2004 279 12588 12597 14711822 10.1074/jbc.M313454200

[B25] Salmon KA Hung S Steffen NR Krupp R Baldi P Hatfield GW Gunsallus R Global gene expression profiling in *Escherichia coli* K12 J Biol Chem 2005 280 15084 15096 10.1074/jbc.M414030200 15699038

[B26] Gao H Wang X Yang ZK Palzkill T Zhou J Probing regulon of ArcA in *Shewanella oneidensis* MR-I by integrated genomic analyses BMC Genomics 2008 9 42 10.1186/1471-2164-9-42 18221523 PMC2262068

[B27] Calderón IL Morales E Caro NJ Chahuán CA Collao B Gil F Villareal JM Ipinza F Mora GC Saavedra CP Response regulator ArcA of *Salmonella enterica* serovar Typhimurium downregulates the expression of OmpD, a porin facilitating uptake of hydrogen peroxide Res Microbiol 2011 162 214 222 10.1016/j.resmic.2010.11.001 21144897

[B28] Morales EH Calderón IL Collao B Gil F Porwollik S McClelland M Saavedra CP Hypochlorous acid and hydrogen peroxide-induced negative regulation of *Salmonella enterica* serovar Typhimurium *ompW* by the response regulator ArcA BMC Microbiol 2012 12 63 10.1186/1471-2180-12-63 22545862 PMC3358236

[B29] Berney M Weilenmann HU Ihssen J Bassin C Egli T Specific growth rate determines the sensitivity of *Escherichia coli* to thermal, UVA, and solar disinfection Appl Environ Microbiol 2006 72 2586 2593 10.1128/AEM.72.4.2586-2593.2006 16597961 PMC1449012

[B30] Santiviago CA Reynolds MM Porwollik S Choi S Long F Andrews-Polymenis HL McClelland M Analysis of pools of targeted *Salmonella* deletion mutants identifies novel genes affecting fitness during competitive infection in mice PLoS Pathog 2009 5 e1000477 10.1371/journal.ppat.1000477 19578432 PMC2698986

[B31] Xia XQ McClelland M Porwollik S Song W Cong X Wang Y WebArrayDB: cross-platform microarray data analysis and public data repository Bioinformatics 2009 25 2425 2429 10.1093/bioinformatics/btp430 19602526 PMC2735672

[B32] Saeed AI Sharov V White J Li J Liang W Bhagabati N Braisted J Klapa M Currier T Thiagarajan M Sturn A Snuffin M Rezantsev A Popov D Ryltsov A Kostukovich E Borisovsky I Liu Z Vinsavich A Trush V Quackenbush J TM4: a free, open-source system for microarray data management and analysis Biotechniques 2003 34 374 378 12613259 10.2144/03342mt01

[B33] Dabney A Storey JD Warnes GR qvalue: Q-value estimation for false discovery rate control R package version 1.26.0. http://www.bioconductor.org/packages/release/bioc/html/qvalue.html

[B34] Subramanian A Tamayo P Mootha VK Mukherjee S Ebert BL Gillette MA Paulovich A Pomeroy SL Golub TR Lander ES Mesirov JP Gene set enrichment analysis: a knowledge-based approach for interpreting genome-wide expression profiles Proc Natl Acad Sci 2005 102 15545 15550 10.1073/pnas.0506580102 16199517 PMC1239896

[B35] Pfaffl MW A new mathematical model for relative quantification in real-time RT-PCR Nucleic Acids Res 2001 29 e45 10.1093/nar/29.9.e45 11328886 PMC55695

[B36] Wang X Gao H Shen Y Weinstock GM Zhou J Palzkill T A high-throuput percentage-of-binding strategy to measure energies in DNA-protein interactions: application to genome-scale discovery Nucleic Acids Res 2001 36 4863 4871 18653527 10.1093/nar/gkn477 PMC2528174

[B37] Jarvik T Smillie C Groisman EA Ochman H Short-term signatures of evolutionary change in the *Salmonella enterica* serovar Typhimurium 14028 genome J Bacteriol 2010 192 560 567 10.1128/JB.01233-09 19897643 PMC2805332

[B38] Turatsinze JV Thomas-Chollier M Defrance M van Helden J Using RSAT to scan genome sequences for transcription factor binding sites and *cis*-regulatory modules Nat Protoc 2008 3 1578 1588 10.1038/nprot.2008.97 18802439

[B39] Echave P Tamarit J Cabiscol E Ros J Novel antioxidant role of alcohol dehydrogenase E from *Escherichia coli* J Biol Chem 2003 278 30193 30198 10.1074/jbc.M304351200 12783863

[B40] Janssen R van der Straaten T van Diepen A van Dissel JT Responses to reactive oxygen intermediates and virulence of *Salmonella* typhimurium Microbes Infect 2003 5 527 534 10.1016/S1286-4579(03)00069-8 12758282

[B41] Wright JA Tötemeyer SS Hautefort I Appia-Ayme C Alston M Danino V Paterson GK Mastroeni P Ménager N Rolfe M Thompson A Ugrinovic S Sait L Humphrey T Northen H Peters SE Maskell DJ Hinton JC Bryant CE Multiple redundant stress resistance mechanisms are induced in *Salmonella enterica* serovar Typhimurium in response to alteration of the intracellular environment via TLR4 signalling Microbiology 2009 155 2919 2929 10.1099/mic.0.030429-0 19542004

[B42] Nelson DL Cox MM Lehninger AL Glycolysis, gluconeogenesis, and the pentose phosphate pathway Lehninger, Principles of Biochemistry 2004 4 New York: WH Freeman 521 559

[B43] Monje-Casas F Jurado J Prieto-Alamo MJ Holmgren A Pueyo C Expression analysis of the *nrdHIEF* operon from *Escherichia coli*. Conditions that trigger the transcript level *in vivo* J Biol Chem 2001 276 18031 18037 10.1074/jbc.M011728200 11278973

[B44] Martin JE Imlay JA The alternative aerobic ribonucleotide reductase of *Escherichia coli*, NrdEF, is a manganese-dependent enzyme that enables cell replication during periods of iron starvation Mol Microbiol 2011 80 319 334 10.1111/j.1365-2958.2011.07593.x 21338418 PMC3097424

[B45] Carmel-Harel O Storz G Roles of glutathione- and thioredoxin-dependent reduction systems in the *Escherichia coli* and *Saccharomyces cerevisiae* responses to oxidative stress Annu Rev Microbiol 2000 54 439 461 10.1146/annurev.micro.54.1.439 11018134

[B46] Imlay JA Fridovich I Assay of metabolic superoxide production in *Escherichia coli* J Biol Chem 1991 266 6957 6965 1849898

[B47] Hébrard M Viala JP Méresse S Barras F Aussel L Redundant hydrogen peroxide scavengers contribute to *Salmonella* virulence and oxidative stress resistance J Bacteriol 2009 191 4605 4614 10.1128/JB.00144-09 19447905 PMC2704729

[B48] Arenas FA Díaz WA Leal CA Pérez-Donoso JM Imlay JA Vásquez CC The *Escherichia coli btuE* gene, encodes a glutathione peroxidase that is induced under oxidative stress conditions Biochem Biophys Res Commun 2010 398 690 694 10.1016/j.bbrc.2010.07.002 20621065 PMC3057470

[B49] Golubeva YA Slauch JM *Salmonella enterica* serovar Typhimurium periplasmic superoxide dismutase sodCI is a member of the PhoPQ regulon and is induced in macrophages J Bacteriol 2006 188 7853 7861 10.1128/JB.00706-06 16980468 PMC1636301

[B50] Ibanez-Ruiz M Robbe-Saule V Hermant D Labrude S Norel F Identification of RpoS (σ^S^)-regulated genes in *Salmonella enterica* serovar Typhimurium J Bacteriol 2000 182 5749 5756 10.1128/JB.182.20.5749-5756.2000 11004173 PMC94696

[B51] Tardat B Touati D Two global regulators repress the anaerobic expression of MnSOD in *Escherichia coli*::Fur (ferric uptake regulation) and Arc (aerobic respiration control) Mol Microbiol 1991 5 455 465 10.1111/j.1365-2958.1991.tb02129.x 2041478

[B52] Alonso-Moraga A Bocanegra A Torres JM López-Barea J Pueyo C Glutathione status and sensitivity to GSH-reacting compounds of *Escherichia coli* strains deficient in glutathione metabolism and/or catalase activity Mol Cell Biochem 1987 73 61 68 3543652 10.1007/BF00229377

[B53] Takemoto T Zhang QM Yonei S Different mechanisms of thioredoxin in its reduced and oxidized forms in defense against hydrogen peroxide in *Escherichia coli* Free Radic Biol Med 1998 24 556 562 10.1016/S0891-5849(97)00287-6 9559867

[B54] Prinz WA Ǻslund F Holmgren A Beckwith J The role of the thioredoxin and glutaredoxin pathways in reducing protein disulfide bonds in the *Escherichia coli* cytoplasm J Biol Chem 1997 272 15661 15667 10.1074/jbc.272.25.15661 9188456

[B55] Henard CA Bourret TJ Song M Vázquez-Torres A Control of the redox balance by the stringent response regulatory protein promotes antioxidant defenses of *Salmonella* J Biol Chem 2010 285 36785 36793 10.1074/jbc.M110.160960 20851888 PMC2978607

[B56] Jordan A Aragall E Gibert I Barbe J Promoter identification and expression analysis of *Salmonella typhimurium* and *Escherichia coli nrdEF* operons encoding one of two class I ribonucleotide reductases present in both bacteria Mol Microbiol 1996 19 777 790 10.1046/j.1365-2958.1996.424950.x 8820648

[B57] Perrenoud A Sauer U Impact of global transcriptional regulation by ArcA, ArcB, Cra, Crp, Cya, Fnr, and Mlc on glucose catabolism in *Escherichia coli* J Bacteriol 2005 187 3171 3179 10.1128/JB.187.9.3171-3179.2005 15838044 PMC1082841

[B58] Messner KR Imlay J The identification of primary sites of superoxide and hydrogen peroxide formation in the aerobic respiratory chain and sulfite reductase complex of Escherichia coli J Biol Chem 1999 274 10119 10128 10.1074/jbc.274.15.10119 10187794

[B59] Korshunov S Imlay JA Two sources of endogenous hydrogen peroxide in *Escherichia coli* Mol Microbiol 2010 75 1389 1401 10.1111/j.1365-2958.2010.07059.x 20149100 PMC3049997

[B60] Uzzau S Figueroa-Bossi N Rubino S Bossi L **Epitope tagging of chromosomal genes in *****Salmonella*** Proc Natl Acad Sci 2001 98 15264 15269 10.1073/pnas.261348198 11742086 PMC65018

