# Supplementary material for: Probing the ArcA regulon under aerobic/ROS conditions in Salmonella enterica serovar Typhimurium
Source: BMC Genomics. 2013 Sep 17;14:626. doi: 10.1186/1471-2164-14-626 (PMC3848847; doi:10.1186/1471-2164-14-626)
Supplement: Additional file 1 — Probing the ArcA regulon under aerobic/ROS conditions in Salmonella enterica serovar Typhimurium. A) Supplementary methods. B) Figure S1: Characterization of the mechanism of ArcA in response to ROS. Measurement of the transcript and protein levels of arcA by qRT-PCR and Western blot, respectively. Determination of CFU/ml in strains 14028s, ΔarcA, ΔarcA::cat/pBR::arcA, and ΔarcA::cat/pBR::arcAD54A, after H2O2 exposure. C) Table S1: Validation of microarray data using qRT-PCR of randomly selected genes. Fold changes are given for the selected genes in response to hydrogen peroxide in the different genetic backgrounds as determined by qRT-PCR and microarray analysis. D) Supplementary references [60]. [file 1471-2164-14-626-S1.doc]

**Additional File 1**

**TITLE: Probing the ArcA regulon under aerobic/ROS conditions in *Salmonella enterica* serovar Typhimurium.**

**Description Additional file 1: A) Supplementary methods. B) Figure S1:** Characterization of the mechanism of ArcA in response to ROS. Measurement of the transcript and protein levels of *arcA* by qRT-PCR and Western blot, respectively. Determination of CFU/ml in strains 14028s, Δ*arcA*, Δ*arcA*::*cat*/pBR::*arcA*, and Δ*arcA*::*cat*/pBR::*arcA*D54A, after H_2_O_2_ exposure. **C) Table S1:** Validation of microarray data using qRT-PCR of randomly selected genes. Fold changes are given for the selected genes in response to hydrogen peroxide in the different genetic backgrounds as determined by qRT-PCR and microarray analysis. **D) Supplementary references.**

1. **Supplemental Methods**

**Real time quantitative RT-PCR of *arcA***

Overnight cultures of strains 14028s and Δ*arcA* were diluted (1:100) and cells were grown to OD_600_ ~ 0.4 as described in methods. At this point, H_2_O_2_ (1 mM) or NaOCl (0.53 mM) was added and cells were grown for 20 min. Control cells received no treatment. Experiments were performed in triplicate on different days. RNA extraction, cDNA generation and qRT-PCR was performed using the primers described in [28], using the conditions described in methods.

**Western blot analysis of ArcA**

A fusion of the sequences encoding the 3xFLAG epitope and the *arcA* gene was constructed as described by Uzzau et al. (2001). Primers were designed with 40-base 5´ extensions corresponding to the 3´-end of the *arcA* coding sequence and to the region immediately downstream of *arcA* to amplify plasmid pSUB11. Primer sequences were 5’ TCACGGCGAAGGTTATCGCTTCTGCGGCGACCTGCAGGATgactacaaagaccatgacgg 3’ (3XFLAG-*arcA*Fw) and 5’ ACAATTTTTATGTAAAAGAGTACGTCATAACGGCAGGTCAcatatgaatatcctccttag 3’ (3XFLAG-*arcA*Rv). The PCR product was used to transform *S*. Typhimurium 14028s carrying plasmid pKD46. The presence of the genetic fusion was confirmed by PCR using primers 5’ GTCGTATTCGTAAGCATTTC 3’ (Comp_flag_arcAF) and 5’ gactaacaaatcccgtgtat 3’ (Comp_flag_arcAR). Strain *arcA*::3xFLAG containing the ArcA-fusion protein was detected by immunoblotting using an anti-FLAG M2 monoclonal antibody (Sigma). Overnight cultures were diluted (1:100) and cells were grown to OD_600_ ~ 0.4 as described in methods. At this point, H_2_O_2_ (1 mM) or NaOCl (0.53mM) was added and cells were grown for 20 min. Control cells received no treatment. Experiments were performed in triplicate on different days. After H_2_O_2_ or NaOCl exposure, cells were centrifuged at 10,000 *x g* for 3 min. Bacterial pellets were suspended in 100 mM Tris-HCl (pH 8.0) and subjected to 3 rounds of sonication of 30 s each. After centrifuging at 13,000 *x g* for 5 min, the supernatant was subjected to SDS-PAGE and size-separated proteins were electroblotted onto nitrocellulose membranes, incubated with anti-FLAG Ab M2 (1:1000 dilution) upon which the FLAG epitope was detected with peroxidase-conjugated anti-mouse IgG and peroxidase activity.

**Complementation of strain Δ*arcA*::*cat***

For complementation studies, the complete *arcA* sequence starting from 180 bp upstream from the start codon (ATG) until the stop codon (TAA) of *arcA* was amplified from the WT strain using primers 5’ CATCGAATTCtacccacgaccaagctaatg 3’(pBR322_arcAF, *Eco*RI site underlined) and 5’ CGCGGATCCTTAATCCTGCAGGTCGCCG 3’ (pBR322_arcAR, *Bam*HI site underlined). The PCR product was digested with *Bam*HI and *Eco*RI and ligated into plasmid pBR322 (Promega), previously digested with the same restriction enzymes. The construction (pBR::*arcA*) was transformed into and maintained in the *S*. Typhimurium *arcA* mutant. Expression of *arcA* by cloning from positions -180 to the stop codon has been shown to be sufficient for its expression [8], therefore avoiding the addition of any external compounds to complement the mutation. To generate the point mutation of *arcA* at position D54, primers pBR322_arcAF with 5’ CCTGGCAGATTGAT**CGC**CATGATCACCAGG 3’ (substitution from D to A underlined) and pBR322_arcAR with 5’ CCTGGTGATCATG**GCG**ATCAATCTGCCAGG 3’ (substitution from D to A underlined) were used to generate overlapping PCR products spanning the whole length of the *arcA* gene from 180 bp upstream from the start codon (ATG) until the stop codon (TAA). The resulting PCR products were used as templates in a second reaction with primers pBR322_arcAF and pBR322_arcAR to generate the mutated *arcA* gene, which was digested and cloned into plasmid pBR322 as described. The construction (pBR::*arcA^D54A^*) was transformed into and maintained in the *S*. Typhimurium *arcA* mutant.

**Bacterial survival after exposure to oxidative stress**

Strains 14028s, Δ*arcA*::cam/pBR, Δ*arcA*::cam/pBR::*arcA* and Δ*arcA*::cam/pBR::*arcA^D54A^* were grown to OD 0.4 and treated with H_2_O_2_ 1 mM as described in methods. Aliquots of cultures were withdrawn at the different time points, diluted and plated in triplicate. Bacterial cultures were enumerated by counting the number of CFU after overnight incubation to determine the bacterial concentrations.

1. **Figure S1**


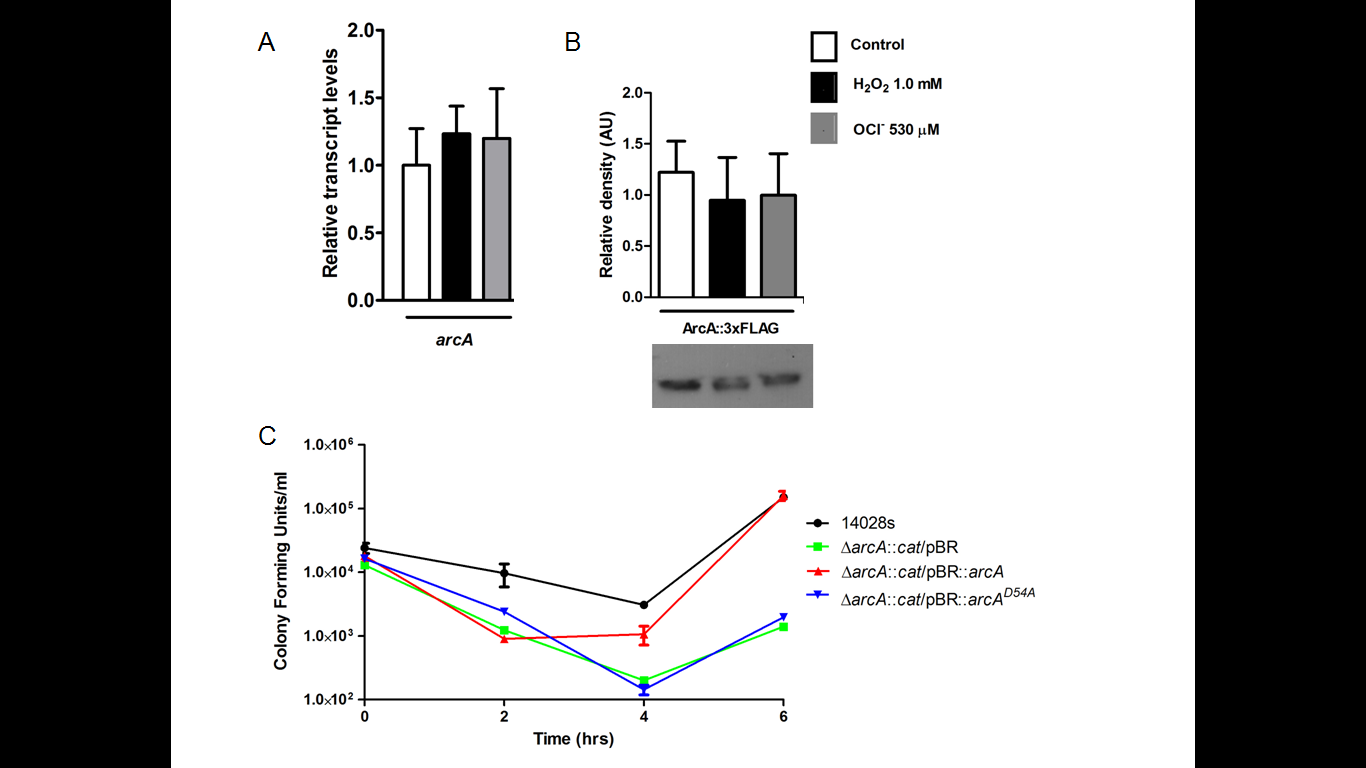


**Figure S1.** Characterization of the mechanism of ArcA in response to ROS. A and B: Strain 14028s grown to OD 0.4 was treated with H_2_O_2_ (1.0 mM) or NaOCl (530 μM) for 20 min and the transcript (A) and protein levels (B) of *arcA* were detected by qRT-PCR and western blot, respectively. Control cells received no treatment. The transcript levels of the 16S rRNA were used for normalization. Protein levels were normalized by selecting three bands from the loading gels. For the western blot one representative experiment is shown. Values represent the average of three independent experiments  ±  SD. C: Strains 14028s, Δ*arcA*::cam/pBR, Δ*arcA*::cam/pBR::*arcA* and Δ*arcA*::cam/pBR::*arcA^D54A^* were grown to OD 0.4 and treated with H_2_O_2_ 1 mM. At the indicated times, the number of CFU/ml were determined for each strain. A representative experiment is shown  ±  SD.

**C. Table S1. Validation of microarray data using qRT-PCR of randomly selected genes**.

| Gene_ID LT2 | Gene name | Primer sequence^A^ | Fold Change H_2_O_2_/control | | | | |  | Fold Change Control | | | Function |
| --- | --- | --- | --- | --- | --- | --- | --- | --- | --- | --- | --- | --- |
|  |  |  | Microarray | |  | qRT-PCR | |  | Microarray |  | qRT-PCR |  |
|  |  |  | 14028s | Δ*arcA* |  | 14028s | Δ*arcA* |  | Δ*arcA*/14028s |  | Δ*arcA*/14028s |  |
| STM0833 | ompX | GGGCGTAGCGAATAAAATGA | 3.65 | -1.14 |  | 2.22 | 1.36 |  | 2.26 |  | 2.19 | outer membrane protein X |
|  |  | GAAGCCATAATCGCTGGTGT |  |  |  |  |  |  |  |  |  |  |
| STM1830 | manX | AGGCGTGCTATTTCTCGTTG | 1.19 | 3.69 |  | -1.96 | 3.13 |  | -1.89 |  | -3.33 | mannose-specific enzyme IIAB |
|  |  | TATAATCATTCGGGCCCATC |  |  |  |  |  |  |  |  |  |  |
| STM1167 | rimJ | GGCGTCTGGCCGATTATTAC | -1.62 | 1.14 |  | -8.24 | 1.18 |  | -1.63 |  | -3.36 | ribosomal-protein-S5-alanine N-acetyltransferase |
|  |  | AGCGGTTAAGGCTTCAAACA |  |  |  |  |  |  |  |  |  |  |
| STM3137 | uxaC | TGAACCCACGTGATAACGAA | -1.79 | 2.35 |  | -6.44 | 2.44 |  | -2.38 |  | -5.61 | glucuronate isomerase |
|  |  | AACCATACGGCTTAGCATCG |  |  |  |  |  |  |  |  |  |  |
| STM4499 | yeeN | GGGACGTAAATGGGCAAATA | 4.32 | 1.55 |  | 5.85 | 1.77 |  | 1.52 |  | 1.87 | hypothetical protein |
|  |  | CGTCAACGTTTCAGCGATAA |  |  |  |  |  |  |  |  |  |  |
| STM0633 | lipA | AGACTTCCGTGGACGTATGG | 2.99 | -1.41 |  | 3.32 | -2.08 |  | 2.04 |  | 3.06 | lipoyl synthase |
|  |  | GCGGCTTGGCTGTAGATACT |  |  |  |  |  |  |  |  |  |  |
| STM2559 | cadA | CGCATAAAGAAGCGGAAGAG | 1.78 | -1.21 |  | 1.53 | -1.24 |  | 1.28 |  | 1.31 | lysine decarboxylase 1 |
|  |  | TTGCGTTAGGCGTCTCTTTT |  |  |  |  |  |  |  |  |  |  |
| STM1750 | tdk | GAAGAGATTCGTGCCGAGAG | 1.46 | -1.59 |  | 1.63 | 1.32 |  | 1.43 |  | 1.70 | thymidine kinase |
|  |  | CACCTGTTCGCCTTCGTTAT |  |  |  |  |  |  |  |  |  |  |

^A^ For each set, first primer listed is the forward primer. Sequence is from 5’ – 3’.

**D. Supplementary references**

60. Uzzau S, Figueroa-Bossi N, Rubino S, Bossi L: **Epitope tagging of chromosomal genes in *Salmonella****. Proc Natl Acad Sci* 2001, **98**: 15264-15269.
